# Appendicoscopy in the identification of a rare case of appendico-sigmoid fistula resulting from appendicitis

**DOI:** 10.1055/a-2598-5036

**Published:** 2025-06-03

**Authors:** Junzhen Li, Chumei Huang, Yingjie Wu, Guinan Liu, Yutao Zhao, Jian Qi, Man Yang

**Affiliations:** 1 543160 Digestive Medicine Center, The Seventh Affiliated Hospital Sun Yat-sen University, Shenzhen, China

**Fig. 1:**
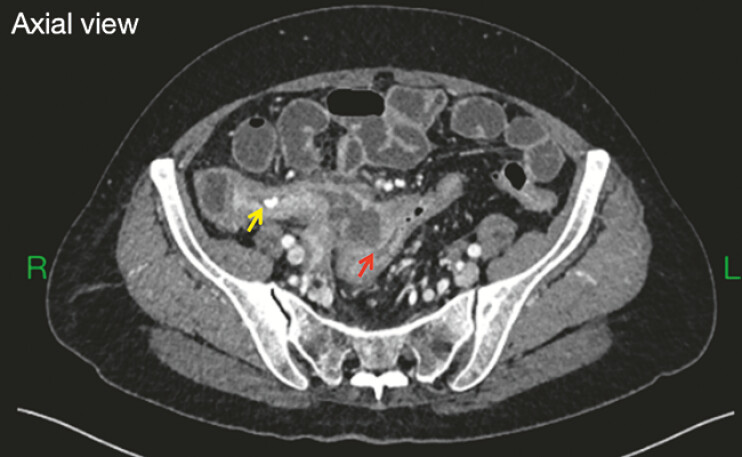
Computed tomography scan showed appendicitis accompanied by a fecalith (yellow arrow) and a local abscess, suggesting possible sigmoid fistula (red arrow).

A 69-year-old woman was admitted due to intermittent lower right abdominal pain for over 20 days. Computed tomography scan revealed appendicitis with associated fecalith and a local abscess communicating with the sigmoid colon, suggesting the possible presence of an appendico-sigmoid fistula ( Fig. 1 ). Endoscopic retrograde appendicitis therapy was performed.

**Fig. 2:**
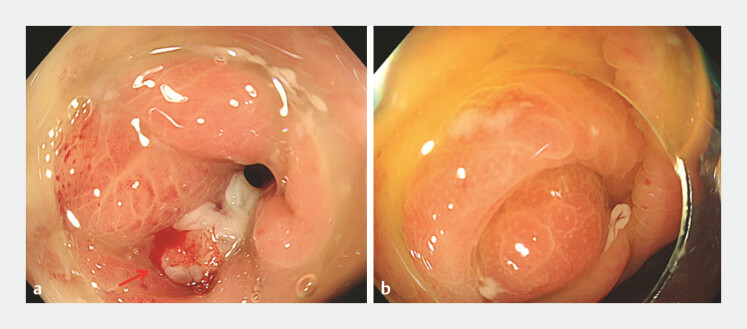
Colonoscopy images. **a** The fistulous opening (red arrow) with white pus in the sigmoid colon. **b** The swollen appendiceal orifice.

**Fig. 3:**
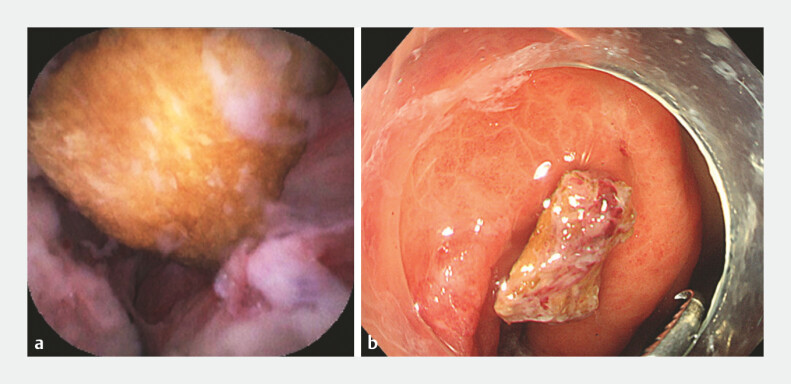
Fecalith removal. **a** The fecalith was detected within the appendix using an appendicoscope. **b** The fecalith was dragged out into the colon cavity for removal.

**Fig. 4:**
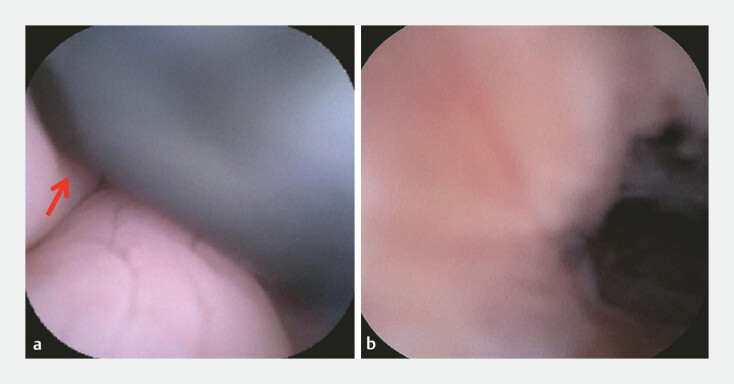
Appendicoscopy images. **a, b** The appendicoscope was inserted further into the colon cavity and the black shaft (red arrow) of the colonoscope was observed, confirming the appendico-sigmoid fistula.

Colonoscopy revealed a fistulous opening with white pus located in the sigmoid colon and a swollen appendiceal orifice ( Fig. 2 ). Appendicoscope (eyeMAX, 9-Fr; Micro-Tech [Nanjing] Co., Ltd., Nanjing, China) was inserted into the appendiceal lumen and revealed a hard yellow impacted fecalith. We removed the fecalith from the appendix with a basket ( Fig. 3 ). The appendicoscope was introduced into the lumen of the appendix under guidance of a guidewire and the appendiceal mucosa exhibited marked congestion and edema. Upon direct inspection, the appendicoscope was inserted into the colon cavity and the black shaft of the colonoscope could be observed, confirming the appendico-sigmoid fistula ( Fig. 4 , Video 1 ). We washed the fistulous tract repeatedly with 0.5% metronidazole. After the treatment, the patient’s abdominal pain improved.

Appendicoscopy identified the appendico-sigmoid fistula resulting from appendicitis.Video 1

To the best of our knowledge, this case represents the first documented endoscopic diagnosis and treatment of an appendico-sigmoid fistula resulting from appendicitis using appendicoscopy under direct visualization.

Endoscopy_UCTN_Code_CCL_1AF_2AG_3AC

